# The effectiveness of cognitive behavioral therapy in patients with motor neuron disease: A systematic review

**DOI:** 10.1097/MD.0000000000043597

**Published:** 2025-07-25

**Authors:** Yue He, Wei Ming, Yinhu Tan, Yang Wang, Mengyao Wang, Hang Li, Zhenzhu Jiao, Yao Hou

**Affiliations:** a School of Nursing, Changchun University of Chinese Medicine, Changchun, Jilin, China; b Department of Orthopedics, The Third Affiliated Hospital of Changchun University of Chinese Medicine, Changchun, Jilin, China.

**Keywords:** acceptance and commitment therapy, amyotrophic lateral sclerosis, cognitive behavioral therapy, mindfulness cognitive therapy, motor neuron disease

## Abstract

**Background:**

Motor neuron disease (MND) is a neurodegenerative disorder that causes progressive loss of motor function. With limited disease-modifying drug therapies, cognitive behavioral therapy (CBT) has emerged as a key nonpharmacological intervention. This systematic review evaluated CBT’s therapeutic potential across clinical domains to inform psychosocial care of patients with MND.

**Methods:**

Comprehensive searches were performed in PubMed, Web of Science, Cochrane Library, and Embase, from inception until February 2025. Two researchers independently screened the literature, extracted data, assessed study quality, and resolved disagreements via consensus or third-party consultation.

**Results:**

Five studies involving 561 patients were included. Compared with conventional care, CBT significantly improves patients’ quality of life and psychological flexibility. However, the effects on caregiver burden and physical health were not statistically significant. CBT modalities included acceptance and commitment therapy, dialectical behavior therapy, rational-emotive behavior therapy, mindfulness cognitive therapy, and metacognitive training. Traditional CBT demonstrated superior efficacy in reducing anxiety and depression compared to acceptance and commitment therapy.

**Conclusion:**

CBT effectively enhances psychological flexibility and quality of life and reduces anxiety and depression in patients with MND. The standardization of outcome measures requires improvement. High-quality randomized controlled trials are needed to further assess CBT’s impact of CBT on caregiver burden and patients’ physical health.

## 1. Introduction

Motor neuron diseases (MND) represent a heterogeneous group of neurodegenerative disorders characterized by selective degeneration of the upper motor neurons (pyramidal cells in the cerebral cortex) and lower motor neurons (anterior horn cells in the spinal cord and brainstem motor nuclei).^[1,2]^ Despite extensive research, the precise etiopathogenesis of these progressive neurological conditions remains unclear. Current classification systems primarily categorize MND into 4 clinical subtypes: amyotrophic lateral sclerosis (ALS), the most prevalent and rapidly progressive form; primary lateral sclerosis; progressive muscular atrophy; and progressive bulbar palsy.^[3]^ Epidemiological data from the 2019 Global Burden of Disease study revealed standardized global prevalence and incidence rates of 3.37 and 0.79 per 100,000 individuals, respectively.^[4]^ Currently, there are no disease-modifying therapies for MND. Pharmacological interventions primarily aim to decelerate disease progression and preserve patient quality of life.^[5,6]^

Cognitive-behavioral therapy, a psychotherapeutic approach integrating cognitive and behavioral principles, targets emotional regulation by modifying maladaptive thought patterns and behaviors.^[7]^ As the predominant psychological intervention for MND, cognitive behavioral therapy (CBT) encompasses multiple modalities including acceptance and commitment therapy (ACT), dialectical behavior therapy, rational-emotive behavior therapy, smindfulness-based cognitive therapy (MBCT), and metacognitive training, each employing distinct therapeutic techniques. Evidence indicates that CBT enhances the quality of life in ALS patients and caregivers with favorable cost-effectiveness.^[8,9]^ Existing studies have demonstrated various applications: ACT-based online interventions alleviate caregiver distress in dementia,^[10]^ dialectical behavior therapy shows efficacy in autism spectrum disorders,^[11]^ rational-emotive behavior therapy improves psychological distress in colorectal cancer patients,^[12]^ mindfulness interventions exhibit high acceptability,^[13]^ and metacognitive training benefits bipolar disorder management.^[14]^ However, CBT’s effectiveness in MND remains inconclusive owing to marked heterogeneity in study quality, discrepancies in outcome measures, and insufficient safety evaluations. This systematic review critically synthesizes existing evidence to inform the optimization of psychological care protocols for patients with MND.

## 2. Methods

This systematic review was conducted following the Cochrane Handbook guidelines^[15]^ and was reported in compliance with PRISMA standards^[16]^ with protocol registration in PROSPERO (CRD42024607527). Heterogeneity in outcome measures precluded quantitative meta-analysis; consequently, the findings were synthesized narratively.

### 2.1. Eligibility criteria

The eligibility criteria were randomized controlled trials (RCTs) with individual/cluster allocation and parallel-group designs, including multi-arm trials. Non-RCT studies, conference abstracts, and non-peer reviewed publications were excluded. Selection followed the Population, Intervention, Comparator, Outcome (PICO) framework without restrictions on sample size, study duration, publication date, or language.

### 2.2. Population (P)

Patients suffering from MND.

### 2.3. Intervention (I)/comparator (C)

The intervention group underwent CBT incorporating mindfulness-based interventions, ACT, and multimodal delivery formats (in-person, digital, and self-administered modalities). Patients in the control group received standard care.

### 2.4. Outcome (O)

According to the criteria established by the authors, the outcome measures focused on evaluating the effectiveness of CBT in patients with MND. These measures primarily encompass the quality of life, psychological flexibility, anxiety and depression symptoms, caregiver burden, and physical health status.

### 2.5. Data sources and search

**Figure 1.**
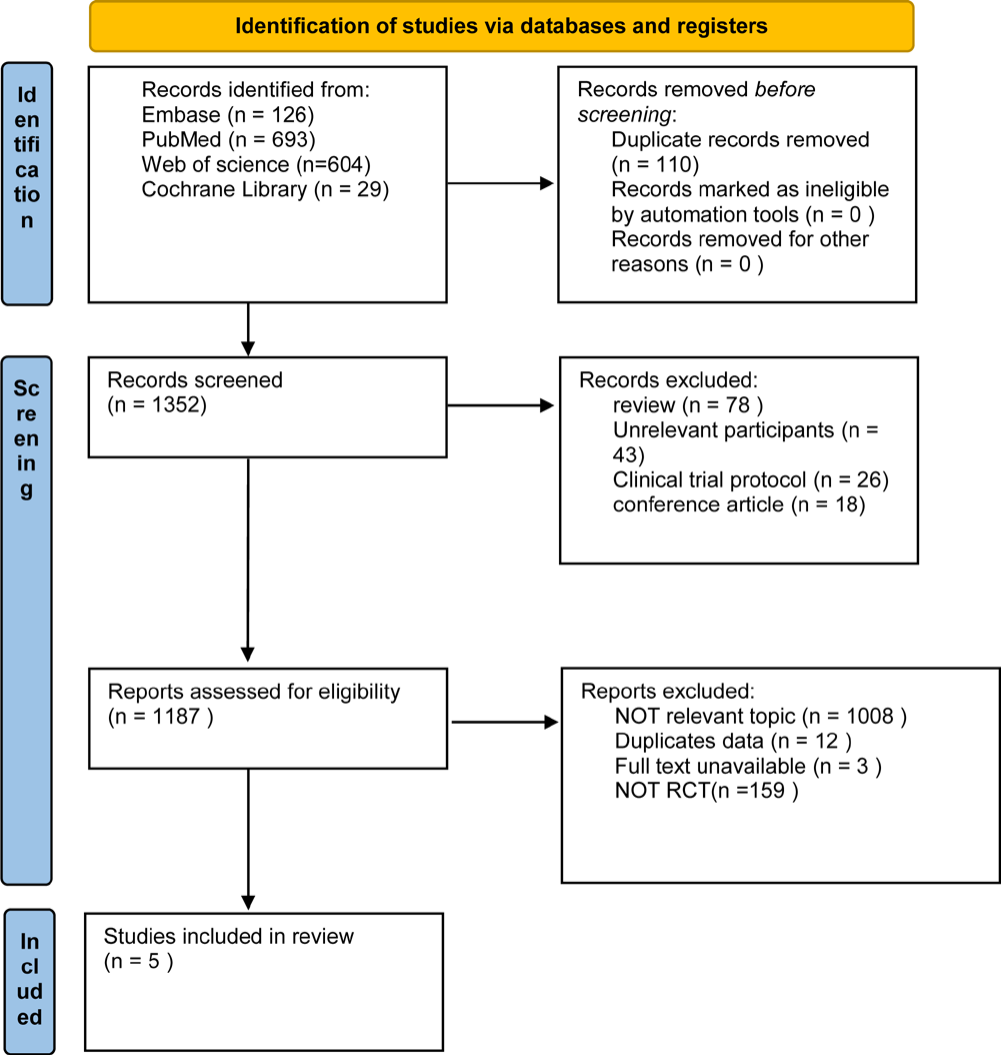
The review process is illustrated. PRISMA (Systematic Reviews and Meta-Analyses) flow diagram.

The PubMed, Web of Science, Cochrane Library, and Embase databases were searched to identify relevant studies that met the inclusion criteria. Detailed search strategies are provided in Appendix 1, Supplemental Digital Content, https://links.lww.com/MD/P543. The search period spanned from the database inception to February 2025. After removing duplicates using NoteExpress software, 2 researchers (H.Y. and T.Y.H.) independently screened the titles and abstracts of all literature for the initial screening. They excluded obviously irrelevant literature, rescreened the full text, and extracted the data. In case of disagreement, they negotiated or consulted a third researcher (W.Y.) for the resolution. The review process is illustrated in Fig. 1.

Two independent reviewers (H.Y. and T. Y. H.) performed blinded quality assessment using the Cochrane Risk of Bias Tool (version 5.1.0),^[17]^ which evaluates 7 methodological domains through “low risk,” “unclear,” or “high risk” classifications. Inter-rater discrepancies were resolved through third-party adjudication (W.Y.) or consensus-based deliberation.

## 3. Results

The systematic search yielded 1452 records, with 5 randomized controlled trials^[18–22]^ meeting eligibility criteria following screening. Methodological quality assessment (Table 1) revealed variable risk of bias across the included studies.

**Table 1:** Literature bias risk table.

	Francesco Pagnini et al^[19]^	Jessica De Wit et al^[20]^	Rebecca L Gould et al^[21]^	Annerieke C et al^[22]^	José Luis Díaz et al^[23]^
Random sequence generation (selection bias)	Low-risk	Low-risk	Low-risk	Low-risk	High-risk
Allocation concealment (selection bias)	High-risk	Unclear	Low-risk	Low-risk	High-risk
Blinding of participants and persons (implementation bias)	Low-risk	Low-risk	Low-risk	Low-risk	Low-risk
Blinding of outcome assessment (detection bias)	Low-risk	Unclear	Unclear	Unclear	Unclear
Incomplete outcome data (dropout bias)	Low-risk	Low-risk	Low-risk	High-risk	Unclear
Selection of reports (reporting bias)	Low-risk	Low-risk	Low-risk	Unclear	Unclear
Other biases	Unclear	Unclear	Unclear	Unclear	Unclear
Grade of quality	B	B	B	B	B

### 3.1. Basic features of literature

Five eligible trials^[18–22]^ enrolled 561 participants (intervention:287; control:274) conducted between 2015 to 2024. Three trials employed single-center designs,^[18,19,22]^ and 2 utilized multicenter frameworks.^[20,21]^ The mean participant age ranged from 54.8 to 66.2 years with female representation spanning 35.8% to 69.4%. The geographical distribution comprised of 4 European nations: the United Kingdom,^[20]^ the Netherlands (n = 2),^[19,21]^ Italy,^[18]^ and Spain.^[22]^ Additional demographic details and the intervention protocols are presented in Tables 2 and 3, respectively.

**Table 2:** Basic characteristics of included studies.

Authors (year)	The state	Study DESIGN	Sample size (patients)		Sample size (caregivers)		Mean age (patients)		Mean age (caregiver)		Proportion of gender (patients, women)	Proportion of gender (caregivers, women)
			Intervention group	Control group	Intervention group	Control group	Intervention group	Control group	Intervention group	Control group		
Francesco Pagnini et al^[19]^	Italy	Two-arm, parallel, single-center RCT	19	19	11	11	63.9 ± 8.3	62.3 ± 8.6	58.1 ± 12.3	62.3 ± 5.7	50%	60%
Jessica De Wit et al^[20]^	Netherlands	Two-arm, parallel, single-center RCT	48	48	26	27	62.3 ± 11.0	62.9 ± 8.9	61.8 ± 10.6	61.3 ± 9.8	35.8%	64.9%
Rebecca L Gould et al^[21]^	Britain	A two-arm, parallel, multicenter RCT	97	94	44	49	61.9 ± 11.4	64.3 ± 10.4	58.2 ± 11.7	60.2 ± 14.2	58%	69%
Annerieke C et al^[22]^	Netherlands	A single-blind, two-arm, parallel, multicenter RCT	10	5	10	5	57.4 ± 50.9	54.8 ± 31.9	57.3 ± 46.2	53.4 ± 34.4	40%	66.7%
José Luis Díaz et al^[23]^	Spain	Single-center RCT	22	16	N/A	N/A	60.6 ± 9.3	66.2 ± 9.5	N/A	N/A	57.9%	N/A

N/A: unable to obtain.

RCTs = randomized controlled trials.

**Table 3:** Specific characteristics of the included study interventions.

Authors (year)	Methods of intervention	Participants of the intervention	Interventions		Duration of intervention	Main outcome measures	Tools for assessment
			Intervention group	Control group			
Francesco Pagnini et al^[19]^	MBCT	Amyotrophic lateral sclerosis (ALS) patients and their primary caregivers	Usual care plus customized online ALS mindfulness sessions with 2 daily practice sessions of 2–3 minutes each.	Usual care	5 weeks, 3 months, and 6 months	Quality of life	①②③
Jessica De Wit et al^[20]^	ACT	Patients with amyotrophic lateral sclerosis (ALS) and progressive muscular atrophy (PMA) and their caregivers	Usual care plus 1 face-to-face contact, 6 online guidance modules and 1 telephone contact. The 6 online tutorials can be completed in 8–12 weeks, and participants can move on to the next module after 1 or 2 weeks, taking approximately 1 hour and 30 minutes to complete each module.	Usual care	3 months, and 6 months	Quality of life	③④⑤
Rebecca L Gould et al^[21]^	ACT	Familial or sporadic amyotrophic lateral sclerosis (ALS); Progressive muscle atrophy (PMA); Or patients with primary lateral sclerosis (PLS) and their caregivers	Usual care + 8 one-to-one ACT sessions, each lasting 1 hour; Meetings are via video call or, in exceptional circumstances, by telephone; The first 6 sessions were held weekly and the last 2 sessions biweekly and were supplemented with online audio materials or CDS.	Usual care	3 months, 6 months and 9 months	Quality of life	②③④⑦⑧⑨
Annerieke C et al^[22]^	CBT	Amyotrophic lateral sclerosis (ALS) patients and caregivers	Usual care + 16 weeks period provided patients with course sessions of 1 hour each at a frequency of 5 to 10 sessions held individually (patient or caregiver) or as a couple (patient and caregiver).	Usual care	4 months, 7 months and 10 months	Quality of life	⑩⑪⑫
José Luis Díaz et al^[23]^	CBT	Patients with amyotrophic lateral sclerosis (ALS)	Usual care + four sessions, each lasting approximately one hour and separated by 15–25 days.	Usual care	11.85 ± 10.85 weeks	Anxiety and depression	②⑤

① ALSSQOL-R: ALS-specific quality of life scale; ② ALS-FRS-R: ALS functional rating scale; ③ ZBI: Zarit Caregiver Burden Inventory; ④ MQOL-R: McGill Quality of Life questionnaire; ⑤ HADS: Hospital Anxiety and Depression Scale; ⑥ AAQ-II: Acceptance and Action Questionnaire-II; ⑦ EQ-5D-5L: Five-level, Five-dimension Health Questionnaire; ⑧ EQ-VAS: EuroQoL visual analogue scale; ⑨ STTS-R: Satisfaction with Therapy and Therapists scale; ⑩ SF-36-MCS: Short Form Health Survey; ⑪ ALSAQ-40-EF: The Emotional functioning subscale of the Amyotrophic Lateral Sclerosis Assessment Questionnaire; ⑫ CSI: caregiver stress index.

ACT = acceptance and commitment therapy, MBCT = mindfulness-based cognitive therapy.

### 3.2. Literature quality assessment

All 5 studies^[18–22]^ demonstrated moderate methodological rigor (grade B). Risk assessment revealed that the selection bias of 4 studies^[18–21]^ was at a low-risk level and that of one study^[22]^ was at a high-risk level. Two studies^[20,21]^ had a low risk of selection bias, 2 studies^[18,22]^ had a high risk of selection bias, and one study^[19]^ had an unclear selection bias. Implementation bias in 5 studies^[18–22]^ was low. The detection bias of one study^[18]^ was at a low risk level, and the detection bias of 4 studies^[19–22]^ was unclear. Three studies^[18–20]^ had a low risk of loss bias, one study^[21]^ had a high risk of loss bias, and one study^[22]^ had an unclear loss bias. The reporting bias of 3 studies^[18–20]^ was at a low risk level, and the reporting bias of 2 studies^[21,22]^ was unclear. Other risks of bias were unknown in 5 studies.^[18–22]^

### 3.3. Literature outcome indicators and main findings

#### 3.3.1. Quality of life

All 5 trials^[18–22]^ demonstrated the therapeutic outcomes of CBT on quality of life (QoL), with 3^[18–20]^ employing multidimensional subscale analyses. Notably, 2 RCTs^[19,20]^ utilizing the McGill Quality of Life questionnaire reported statistically significant QoL improvements through ACT in patients with ALS. A trial^[20]^ incorporating the EuroQoL 5-Dimension 5-Level instrument found comparable QoL decline between groups. Trial^[19]^ with the ALS-specific QoL scale revealed attenuated deterioration in the intervention group at 6-month follow-up. A trial^[21]^ employing the SF-36 demonstrated a slower decline in QoL in CBT recipients over time (*P* < .05).

#### 3.3.2. Flexibility of mind

A trial^[20]^ specifically assessed the impact of acceptance and ACT on psychological flexibility in patients with MND using the Acceptance and Action Questionnaire-II (AAQ-II), where higher scores indicate diminished psychological flexibility and elevated experiential avoidance. Post-intervention analysis revealed a significant reduction in AAQ-II scores in the ACT group. As the core mechanism of ACT, enhanced psychological flexibility demonstrated predictive validity for both quality of life improvement and negative emotion mitigation, enabling value-driven behavioral adaptation through increased cognitive reframing capacity.

#### 3.3.3. Anxiety and depression

Three trials^[19,20,22]^ employing the Hospital Anxiety and Depression Scale demonstrated anxiolytic and antidepressant effects in patients with ALS, with intervention groups showing progressive symptom reduction versus control group deterioration. Temporal analysis revealed trial^[20]^ maximum symptom alleviation at the 6-month follow-up, with partial rebound at 9 months. Trial^[19]^ peak improvement at 3-month assessment, followed by gradual attenuation over 6 months. Trial^[22]^ conventional CBT protocols have achieved sustained post-intervention symptom reduction.

#### 3.3.4. Caregiver burden

Four studies^[18–21]^ investigated the impact of CBT on caregiver burden metrics, and 3 trials^[18–20]^ employing the Zarit Burden Inventory demonstrated nonsignificant burden escalation. A trial^[21]^ utilizing the Caregiver Strain Index revealed differential trajectories: control group burden increased versus stable intervention group levels.

#### 3.3.5. Physical health status

Three trials^[18,20,22]^ that assessed physical function using the Amyotrophic Lateral Sclerosis Functional Rating Scale revealed progressive motor function deterioration across cohorts. Notably, Gould et al^[20]^ employed a parallel assessment with the EuroQoL Visual Analog Scale and demonstrated time-dependent outcomes: significant improvement in global health status after a post-6-month ACT intervention. Attenuated therapeutic effect at 9-month follow-up.

## 4. Discussion

This systematic review demonstrated that CBT demonstrates therapeutic efficacy in enhancing psychological flexibility, quality of life, and affective symptom management, although it demonstrates limited efficacy in mitigating caregiver burden and physical health deterioration. These findings align with the established CBT mechanisms observed in populations with chronic disease,^[7,23,24]^ confirming its transdiagnostic therapeutic value.

The reviewed CBT variants comprised 3 distinct therapeutic frameworks: MBCT emphasizing present-moment awareness, and nonjudgmental acceptance.^[18]^ Traditional CBT protocols target cognitive restructuring through negative thought identification and modification.^[21,22]^ ACT promotes psychological flexibility via value-driven behavioral activation.^[19,20]^ Comparative analysis demonstrated that MBCT and ACT protocols exhibit broader clinical applicability for sustained psychological outcomes and relapse prevention than conventional CBT approaches.

This synthesis demonstrates that all 3 CBT modalities, traditional CBT, MBCT, and ACT, are effective in enhancing the quality of life of patients with MND. Comparative analysis revealed that traditional CBT exhibits superior therapeutic performance in ameliorating affective symptoms compared to ACT. Progressive functional deterioration (e.g., dysphagia and respiratory compromise) inherently limits engagement in structured psychotherapeutic protocols, as evidenced by existing pathophysiological studies.^[25]^ Therapeutic applicability analysis indicated that Traditional CBT demonstrates optimal efficacy during early to mid-disease stages, where preserved motor functionality and cognitive reserve enable rapid symptom mitigation through structured cognitive restructuring and behavioral activation techniques. ACT is particularly advantageous in advanced disease management as it addresses the psychological demands of irreversible functional decline through value-based psychological adaptation frameworks.

The reviewed cognitive behavioral interventions demonstrated modality-specific implementation frameworks and outcomes. Traditional CBT, administered by certified psychologists through structured six-module protocols (diagnosis acceptance, emotional regulation, autonomy maintenance, social support mobilization, future-oriented coping, and activity preservation) delivered in weekly 60-minute sessions, effectively enhanced QoL while mitigating caregiver burden in patients.^[21,22]^ MBCT employed a digital psychoeducation platform featuring thematic weekly webinars coupled with caregiver-assisted daily mindfulness exercises (video tutorials and written materials), demonstrating particular efficacy in alleviating affective symptoms.^[18]^ Conversely, ACT implementations, which typically involve clinician-led value clarification sessions supplemented with asynchronous digital reinforcement (e.g., Gould et al’s^[20]^ hybrid model integrating teleconsultations with audio/CD materials), showed paradoxical outcomes: improved physical health parameters alongside exacerbated caregiver strain,^[19,20]^ potentially attributable to the bidirectional caregiver–patient distress cycle inherent in neurodegenerative symptom management.^[26–28]^

Empirical evidence establishes psychological flexibility as a critical determinant of life, with meta-analytic data demonstrating robust correlations between adaptive cognitive-emotional regulation capacities and enhanced life satisfaction/mental health outcomes (*R* = 0.54–0.61, *P* < .001).^[29,30]^ In MND trajectories, progressive functional decline coupled with treatment-related distress precipitates measurable reduction in psychological flexibility. Notably, while ACT enhances this construct during the initial intervention phases, longitudinal analysis reveals temporal dynamics: trial^[20]^ documented maximal AAQ-II score reduction at the 6-month follow-up (AAQ-II: mean = 16.8–15.5), with stabilization thereafter at 9-month (AAQ-II: mean = 15.5–15.2), suggesting diminishing returns with prolonged intervention. These findings underscore the necessity of chronotherapeutic optimization in ACT protocols to maximize psychological flexibility gains during the critical therapeutic window.

This systematic review substantiates the therapeutic efficacy of CBT in ameliorating the core clinical manifestations of MND, including deficits in quality of life, psychological rigidity, and affective disturbances, although the longitudinal therapeutic sustainability remains inadequately characterized. Current evidence is constrained by methodological limitations including suboptimal intervention protocols and ill-defined temporal parameters. To address these gaps, future research imperatives include (1) confirmatory randomized controlled trials with standardized CBT dosing regimens, (2) development of precision psychiatry frameworks integrating multimodal therapeutic approaches tailored to disease-stage-specific pathophenotypes, and (3) implementation of AI-driven adaptive algorithms that enable real-time protocol optimization to prevent therapeutic overexposure while maintaining intervention fidelity.

## 5. Limitations

This systematic review was subjected to several methodological constraints. First, while implementing a comprehensive search strategy, the exclusion of non-English publications and gray literature from database inception through February 2025 may introduce selection bias. Second, the limited sample size (n = 5 trials) heightened vulnerability to single-study dominance effects, compounded by inherent challenges in conducting longitudinal neuropsychological interventions given the epidemiological characteristics of MND, notably low incidence rates (0.79/100,000), and rapid disease progression that truncates therapeutic windows. Furthermore, CBT’s mechanism of action of CBT typically necessitates longitudinal protocols spanning weeks to months for measurable neurobehavioral modulation, a requirement that is frequently unattainable in MND populations. Finally, inadequate statistical power owing to small cohort sizes and insufficient accounting for phenotypic heterogeneity underscores the need for future large-scale multicenter trials incorporating stratified subgroup analyses.

## 6. Conclusion

Emerging evidence substantiates the therapeutic efficacy of CBT in enhancing psychological flexibility, QoL, and affective symptom management in patients with MND, although it has a limited impact on caregiver burden indices and general health metrics. As a non-pharmacological intervention within an incurable neurodegenerative context, where care prioritizes symptom palliation and QoL optimization, CBT provides a critical modality for addressing psychosocial sequelae by targeting disease-related cognitive distortions. This systematic review highlights CBT’s viability as an adjunctive psychosocial support mechanism; however, methodological limitations, including restricted sample sizes (n = 5 studies), heterogeneous outcome measures, and unstandardized assessment protocols, preclude quantitative synthesis. Research imperatives include large-scale RCTs employing standardized assessment batteries, with particular emphasis on phenotype-stratified analyses (bulbar vs spinal onset variants), and dual-perspective evaluations incorporating patient–caregiver dyad assessments to comprehensively characterize intervention value.

## Author contributions

**Data curation:** Wei Ming.

**Formal analysis:** Yue He.

**Investigation:** Yue He.

**Methodology:** Wei Ming, Yang Wang.

**Software:** Yinhu Tan, Mengyao Wang.

**Supervision:** Hang Li, Zhenzhu Jiao, Yao Hou.

**Writing – original draft:** Yue He, Hang Li.

**Writing – review & editing:** Yue He, Wei Ming, Yinhu Tan, Yang Wang, Mengyao Wang, Zhenzhu Jiao, Yao Hou.

## Supplementary Material
