# Supplementary material for: The effectiveness of cognitive behavioral therapy in patients with motor neuron disease: A systematic review
Source: Medicine (Baltimore). 2025 Jul 25;104(30):e43597. doi: 10.1097/MD.0000000000043597 (PMC12303493; doi:10.1097/MD.0000000000043597)
Supplement: Supplementary file 1 [file medi-104-e43597-s001.docx]

**SEARCH STRATEGIES**

**PUBMED Literature Search Strategy.**

| Search No. | Search Terms |
| --- | --- |
| Disease | |
| #1 | Motor Neuron Diseases[Title/Abstract] OR Neuron Disease, Motor[Title/Abstract] OR Neuron Diseases, Motor[Title/Abstract] OR Motor System Disease[Title/Abstract] OR Motor System Diseases[Title/Abstract] OR Anterior Horn Cell Disease[Title/Abstract] OR Lateral Sclerosis[Title/Abstract] OR Lateral Scleroses[Title/Abstract] OR Scleroses, Lateral[Title/Abstract] OR Sclerosis, Lateral[Title/Abstract] OR Primary Lateral Sclerosis[Title/Abstract] OR Lateral Scleroses, Primary[Title/Abstract] OR Lateral Sclerosis, Primary[Title/Abstract] OR Primary Lateral Scleroses[Title/Abstract] OR Scleroses, Primary Lateral[Title/Abstract] OR Sclerosis, Primary Lateral[Title/Abstract] OR Motor Neuron Disease, Lower[Title/Abstract] OR Lower Motor Neuron Disease[Title/Abstract] OR Motor Neuron Disease, Upper[Title/Abstract] OR Upper Motor Neuron Disease[Title/Abstract] OR Motor Neuron Disease, Secondary[Title/Abstract] OR Secondary Motor Neuron Disease[Title/Abstract] OR Familial Motor Neuron Disease[Title/Abstract] OR Motor Neuron Disease, Familial[Title/Abstract] OR amyotrophic lateral sclerosis[Title/Abstract] OR Gehrig Disease[Title/Abstract] |
| #2 | "Motor Neuron Disease"[Mesh] |
| #3 | #1 OR #2 |
| Management | |
| #4 | Behavioral Therapies, Cognitive[Title/Abstract] OR Behavioral Therapy, Cognitive[Title/Abstract] OR Cognitive Behavioral Therapies[Title/Abstract] OR Therapies, Cognitive Behavioral[Title/Abstract] OR Therapy, Cognitive Behavioral[Title/Abstract] OR Cognition Therapy[Title/Abstract] OR Cognition Therapies[Title/Abstract] OR Therapies, Cognition[Title/Abstract] OR Therapy, Cognitive Behavior[Title/Abstract] OR Behavior Therapies, Cognitive[Title/Abstract] OR Cognitive Behavior Therapies[Title/Abstract] OR Therapies, Cognitive Behavior[Title/Abstract] OR Therapy, Cognition[Title/Abstract] OR Behavior Therapy, Cognitive[Title/Abstract] OR Cognitive Behavior Therapy[Title/Abstract] OR Cognitive Psychotherapy[Title/Abstract] OR Cognitive Psychotherapies[Title/Abstract] OR Psychotherapies, Cognitive[Title/Abstract] OR Psychotherapy, Cognitive[Title/Abstract] OR Therapy, Cognitive[Title/Abstract] OR Cognitive Therapies[Title/Abstract] OR Therapies, Cognitive[Title/Abstract] OR Cognitive Behaviour Therapy[Title/Abstract] OR Behaviour Therapies, Cognitive[Title/Abstract] OR Behaviour Therapy, Cognitive[Title/Abstract] OR Cognitive Behaviour Therapies[Title/Abstract] OR Therapies, Cognitive Behaviour[Title/Abstract] OR Therapy, Cognitive Behaviour[Title/Abstract] OR Cognitive Therapy[Title/Abstract] OR Acceptance and Commitment Therapy[Title/Abstract] OR Mindfulness-Based Cognitive Therapy[Title/Abstract] |
| #5 | Acceptance and Commitment Therapy[Title/Abstract] OR Mindfulness-Based Cognitive Therapy[Title/Abstract] |
| #6 | "Cognitive Behavioral Therapy"[Mesh] |
| #7 | #4 OR #5 OR #6 |
| Total | |
| #8 | #3 AND #7 |

**WEB OF SCIENCE Literature Search Strategy.**

| Search No. | Search Terms |
| --- | --- |
| Disease | |
| #1 | Motor Neuron Diseases OR Neuron Disease, Motor OR Neuron Diseases, Motor OR Motor System Disease OR Motor System Diseases OR Anterior Horn Cell Disease OR Lateral Sclerosis OR Lateral sclerosis OR sclerosis, Lateral OR Sclerosis, Lateral OR Primary Lateral Sclerosis OR Lateral sclerosis, Primary OR Lateral Sclerosis, Primary OR Primary Lateral sclerosis OR sclerosis, Primary Lateral OR Sclerosis, Primary Lateral OR Motor Neuron Disease, Lower OR Lower Motor Neuron Disease OR Motor Neuron Disease, Upper OR Upper Motor Neuron Disease OR Motor Neuron Disease, Secondary OR Secondary Motor Neuron Disease OR Familial Motor Neuron Disease OR Motor Neuron Disease, Familial OR amyotrophic lateral sclerosis OR Gehrig Disease (Topic) |
| Management | |
| #2 | Behavioral Therapies, Cognitive OR Behavioral Therapy, Cognitive OR Cognitive Behavioral Therapies OR Therapies, Cognitive Behavioral OR Therapy, Cognitive Behavioral OR Cognition Therapy OR Cognition Therapies OR Therapies, Cognition OR Therapy, Cognitive Behavior OR Behavior Therapies, Cognitive OR Cognitive Behavior Therapies OR Therapies, Cognitive Behavior OR Therapy, Cognition OR Behavior Therapy, Cognitive OR Cognitive Behavior Therapy OR Cognitive Psychotherapy OR Cognitive psychotherapist OR psychotherapist, Cognitive OR Psychotherapy, Cognitive OR Therapy, Cognitive OR Cognitive Therapies OR Therapies, Cognitive OR Cognitive Behaviour Therapy OR Behaviour Therapies, Cognitive OR Behaviour Therapy, Cognitive OR Cognitive Behaviour Therapies OR Therapies, Cognitive Behaviour OR Therapy, Cognitive Behaviour OR Cognitive Therapy OR Acceptance and Commitment Therapy OR Mindfulness-Based Cognitive Therapy (Topic) |
| Total | |
| #3 | #1 AND #2 |

**EMBASE Literature Search Strategy.**

| Search No. | Search Terms |
| --- | --- |
| Disease | |
| #1 | 'motor neuron diseases':ab,ti OR 'neuron disease, motor':ab,ti OR 'neuron diseases, motor':ab,ti OR 'motor system disease':ab,ti OR 'motor system diseases':ab,ti OR 'anterior horn cell disease':ab,ti OR 'lateral sclerosis':ab,ti OR 'lateral scleroses':ab,ti OR 'scleroses, lateral':ab,ti OR 'sclerosis, lateral':ab,ti OR 'primary lateral sclerosis':ab,ti OR 'lateral scleroses, primary':ab,ti OR 'lateral sclerosis, primary':ab,ti OR 'primary lateral scleroses':ab,ti OR 'scleroses, primary lateral':ab,ti OR 'sclerosis, primary lateral':ab,ti OR 'motor neuron disease, lower':ab,ti OR 'lower motor neuron disease':ab,ti OR 'motor neuron disease, upper':ab,ti OR 'upper motor neuron disease':ab,ti OR 'motor neuron disease, secondary':ab,ti OR 'secondary motor neuron disease':ab,ti OR 'familial motor neuron disease':ab,ti OR 'motor neuron disease, familial':ab,ti OR 'amyotrophic lateral sclerosis':ab,ti OR 'gehrig disease':ab,ti |
| Management | |
| #2 | 'behavioral therapies, cognitive':ti,ab,kw OR 'behavioral therapy, cognitive':ti,ab,kw OR 'cognitive behavioral therapies':ti,ab,kw OR 'therapies, cognitive behavioral':ti,ab,kw OR 'therapy, cognitive behavioral':ti,ab,kw OR 'cognition therapy':ti,ab,kw OR 'cognition therapies':ti,ab,kw OR 'therapies, cognition':ti,ab,kw OR 'therapy, cognitive behavior':ti,ab,kw OR 'behavior therapies, cognitive':ti,ab,kw OR 'cognitive behavior therapies':ti,ab,kw OR 'therapies, cognitive behavior':ti,ab,kw OR 'therapy, cognition':ti,ab,kw OR 'behavior therapy, cognitive':ti,ab,kw OR 'cognitive behavior therapy':ti,ab,kw OR 'cognitive psychotherapy':ti,ab,kw OR 'cognitive psychotherapies':ti,ab,kw OR 'psychotherapies, cognitive':ti,ab,kw OR 'psychotherapy, cognitive':ti,ab,kw OR 'therapy, cognitive':ti,ab,kw OR 'cognitive therapies':ti,ab,kw OR 'therapies, cognitive':ti,ab,kw OR 'cognitive behaviour therapy':ti,ab,kw OR 'behaviour therapies, cognitive':ti,ab,kw OR 'behaviour therapy, cognitive':ti,ab,kw OR 'cognitive behaviour therapies':ti,ab,kw OR 'therapies, cognitive behaviour':ti,ab,kw OR 'therapy, cognitive behaviour':ti,ab,kw OR 'cognitive therapy':ti,ab,kw OR 'acceptance and commitment therapy':ti,ab,kw OR 'mindfulness-based cognitive therapy':ti,ab,kw |
| Total | |
| #3 | #1 AND #2 |

**COCHRANE LIBRARY Literature Search Strategy.**

| Search No. | Search Terms |
| --- | --- |
| Disease | |
| #1 | (Motor Neuron Diseases OR Neuron Disease, Motor OR Neuron Diseases, Motor OR Motor System Disease OR Motor System Diseases OR Anterior Horn Cell Disease OR Lateral Sclerosis OR Lateral Scleroses OR Scleroses, Lateral OR Sclerosis, Lateral OR Primary Lateral Sclerosis OR Lateral Scleroses, Primary OR Lateral Sclerosis, Primary OR Primary Lateral Scleroses OR Scleroses, Primary Lateral OR Sclerosis, Primary Lateral OR Motor Neuron Disease, Lower OR Lower Motor Neuron Disease OR Motor Neuron Disease, Upper OR Upper Motor Neuron Disease OR Motor Neuron Disease, Secondary OR Secondary Motor Neuron Disease OR Familial Motor Neuron Disease OR Motor Neuron Disease, Familial OR amyotrophic lateral sclerosis OR Gehrig Disease):ti,ab,kw |
| Management | |
| #2 | (Behavioral Therapies, Cognitive OR Behavioral Therapy, Cognitive OR Cognitive Behavioral Therapies OR Therapies, Cognitive Behavioral OR Therapy, Cognitive Behavioral OR Cognition Therapy OR Cognition Therapies OR Therapies, Cognition OR Therapy, Cognitive Behavior OR Behavior Therapies, Cognitive OR Cognitive Behavior Therapies OR Therapies, Cognitive Behavior OR Therapy, Cognition OR Behavior Therapy, Cognitive OR Cognitive Behavior Therapy OR Cognitive Psychotherapy OR Cognitive Psychotherapies OR Psychotherapies, Cognitive OR Psychotherapy, Cognitive OR Therapy, Cognitive OR Cognitive Therapies OR Therapies, Cognitive OR Cognitive Behaviour Therapy OR Behaviour Therapies, Cognitive OR Behaviour Therapy, Cognitive OR Cognitive Behaviour Therapies OR Therapies, Cognitive Behaviour OR Therapy, Cognitive Behaviour OR Cognitive Therapy OR Acceptance and Commitment Therapy OR Mindfulness-Based Cognitive Therapy):ti,ab,kw |
| Total | |
| #3 | #1 AND #2 |
